# Shape of Testosterone

**DOI:** 10.1021/acs.jpclett.1c01743

**Published:** 2021-07-20

**Authors:** Iker León, Elena R. Alonso, Santiago Mata, José L. Alonso

**Affiliations:** † Instituto Biofisika (UPV/EHU, CSIC), University of the Basque Country, 48940 Leioa, Spain; ‡ Departamento de Química Física, Facultad de Ciencia y Tecnología, Universidad del País Vasco, Barrio Sarriena s/n, 48940 Leioa, Spain; § Grupo de Espectrocopía Molecular (GEM), Edificio Quifima, Laboratorios de Espectroscopia y Bioespectroscopia, Unidad Asociada CSIC, Parque Científico UVa, Universidad de Valladolid, 47011 Valladolid, Spain

## Abstract

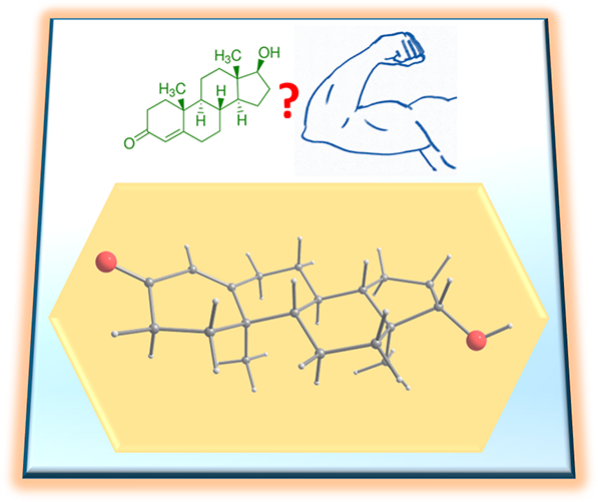

We have successfully characterized the structure of testosterone, one of the essential steroids, through high-resolution rotational spectroscopy. A single conformer has been detected, and a total of 404 transitions have been fitted, allowing a precise determination of the rotational constants. It allowed us to unravel that the isolated structure of testosterone adopts an extended disposition. The results obtained in this work highlight how using laser ablation techniques in combination with Fourier transform microwave techniques allow the study of large biomolecules or common pharmaceuticals. It is an important step toward studying relevant biomolecules and developing new analytical techniques with unprecedented sensitivity and resolution.

Testosterone (17β-hydroxyandrost-4-en-3-one, mp = 155 °C), shown in Scheme 1, is one of the most relevant steroids.^1,2^ It is a sex hormone that plays an essential role in the body. It regulates sex drive (libido), fat distribution, body hair, bone mass, muscle mass, and red blood cells in humans. It also plays a crucial role in sperm production and developing male reproductive tissues such as the testes and prostate. It is biosynthesized from cholesterol through a series of steps, and a small amount of circulating testosterone is transformed into estradiol, a form of estrogen. Though it is also present in females to a lesser extent, they are more sensitive to it. As men age, the testosterone levels decrease, producing less estradiol. Thus, changes attributed to testosterone deficiency might be partly or entirely due to the accompanying decline in estradiol. Additionally, testosterone is also used as a medication for breast cancer treatment in women and low testosterone levels in men.

**Scheme 1:**
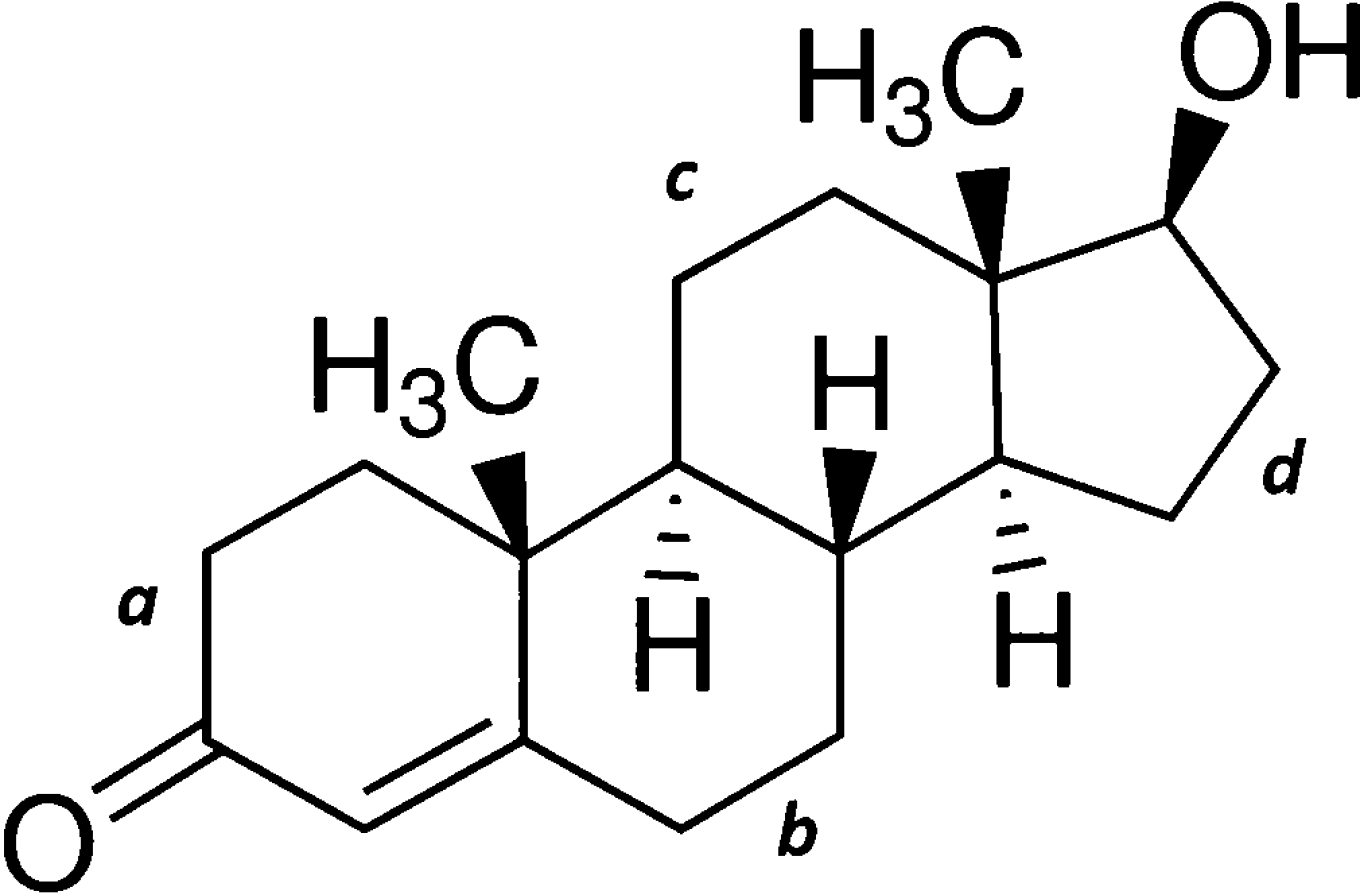
Chemical Structure of Testosterone The labels *a–d* are used to reference the different rings in refs (5) and (6).

It is well-known that there is a direct relationship between a simple or macromolecule’s structure and its particular function or properties.^3,4^ Obtaining the precise structure of testosterone is therefore needed to understand its activity. So far, nuclear magnetic resonance (NMR) spectroscopic studies have been conducted.^5,6^ These high-resolution studies conducted under the solid-state ^13^C NMR technique show the existence of two species. However, the authors highlight how care should be taken when transferring conformational information from crystalline to the solution states,^6^ since the conformational landscape of a molecule can be perturbed due to the surrounding solvent effects. An effective way of removing such effects is the use of gas-phase spectroscopic techniques, most notably supersonic expansions. The absence of any solvent provides the conformational panorama unbiased by perturbing agents.^7−10^

A significant limitation occurring in gas-phase studies is the size of a molecule: as growing molecular size reduces the vapor pressure, i.e., the larger the molecular size and the lower the vapor pressure, more complex organic molecules cannot be driven into the gas phase. It is essential for biomolecules that cannot be studied using conventional heating methods due to their thermolability and low vapor pressure. One exception is estradiol, a vital steroid hormone. Its rotational spectrum has been recently reported, and three conformers have been characterized.^11^ In a first attempt, we tried to measure the rotational spectrum of testosterone through heating, but no spectral signature was obtained. This problem can be overcome by using laser ablation techniques, which have proven to be successful for many biomolecules.^12,13^ Using this methodology, we were successful in obtaining the first rotational spectrum of testosterone.

We used our laser ablation chirped-pulse Fourier transform microwave (LA-CP-FTMW) spectrometer^14,15^ to obtain the microwave spectrum of testosterone in the 1.5–6.5 GHz frequency range as shown in Figure 1a. Obtaining the laser-ablated rotational spectrum of such a large biomolecule is not easy and requires careful control of the experimental parameters as well as its fragmentation to be minimized.^12^ As can be seen, the spectrum shows very intense rotational lines. Initially, lines corresponding to known photofragment species and water clusters were identified and removed.^14,16,17^ The remaining rotational spectrum still showed many rotational transitions, subsequently attributed to testosterone. At first glance, the characteristic pattern of an *a*-type R-branch progression arising from a dominant rotameric species (see Figure 1a) was quickly identified. The first set of rotational constants determined from a rigid rotor analysis^18^ helped us quickly locate *b*- and *c*-type transitions with new predictions. A total of 404 rotational transitions were assigned and measured (Table S1 of the Supporting Information, SI), allowing us to determine very accurate values of the rotational constants. Figure 1b shows excellent matching between experimental and simulated spectra using the fitted values, which are listed in the first column of Table 1. After the rotational lines of this rotamer were removed, no significant signals remained unassigned in the spectrum. Therefore, no spectral searches for other conformational candidates were conducted.

**Figure 1:**
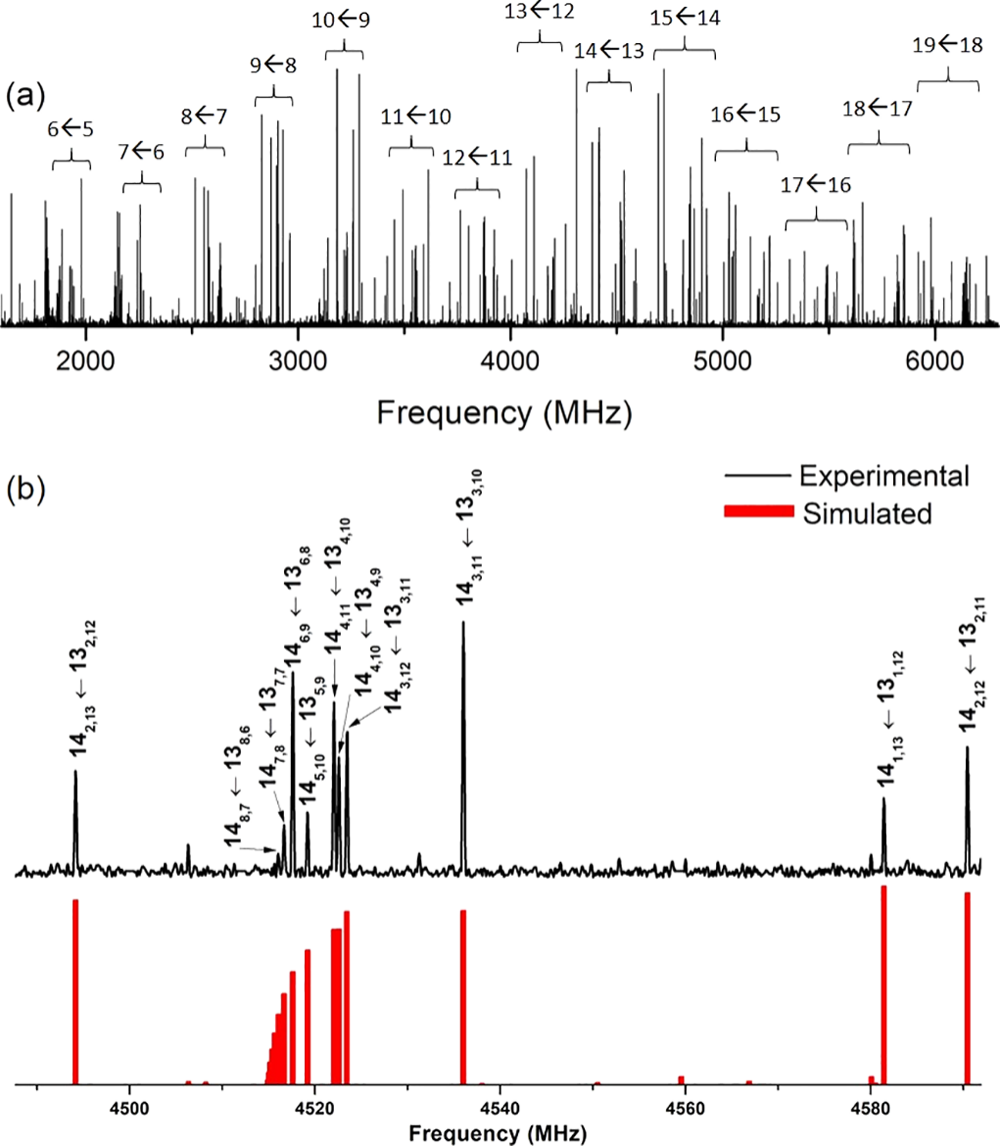
(a) Broadband spectrum of testosterone in the 1.5–6.5 GHz frequency region using the LA-CP-FTMW spectrometer, highlighting the *a*-type R-branch progressions (J' ← J) originated by a single conformer of testosterone. (b) A comparison between a selected range of the experimental spectrum with the simulated one, with selected rotational transitions (J'´_K'a,K'c_ ← J_Ka,Kc_). As can be seen, there is an excellent agreement between theory and experiment.

**Table 1:** Experimental Spectroscopic Parameters for Testosterone’s Detected Conformer along with Plausible Configurations I and II^a^ Calculated at the B3LYP-GD3/6-311++G(d,p) Level of Theory

		configuration I			configuration II		
	experimental	1	2	3	4	5	6
*A*^b^	785.3463(11)^h^	789	787	788	667	665	666
*B*	168.66869(21)	168	168	169	182	182	182
*C*	153.72059(18)	153	153	153	171	171	172
|μ_*a*_|	observed	3.4	3.8	4.9	3.0	3.6	4.4
|μ_*b*_|	observed	0.8	0.7	0.6	0.5	1.2	0.9
|μ_*c*_|	observed	1.1	2.8	0.8	2.1	3.6	1.4
σ^c^	19.0						
*N*^d^	404						
Δ*E*^e^		0	24	73	492	523	556
Δ*E*_ZPE_^f^		0	45	64	546	595	598
Δ*G*^g^		0	67	62	596	664	642

a See text for details.

b *A*, *B*, and *C* represent the rotational constants (in MHz); μ_*a*_, μ_*b*_, and μ_*c*_ are the electric dipole moment components (in D).

c RMS deviation of the fit (in kHz).

d Number of measured transitions.

e Energies (in cm^–1^) relative to the global minimum calculated at the B3LYP-GD3BJ/6-311++G(d,p) level of theory.

f Energies (in cm^–1^) relative to the global minimum, taking into account the zero-point energy (ZPE), calculated at the B3LYP-GD3BJ/6-311++G(d,p) level of theory.

g Gibbs energies (in cm^–1^) relative to the global minimum calculated at 298 K at the B3LYP-GD3BJ/6-311++G(d,p) level of theory.

h Standard error in parentheses in units of the last digit.

To help identify the observed rotamer of testosterone, we performed a conformational search using molecular mechanics methods. A total of 8 molecular structures were screened within an energy window of 2500 cm^–1^. These structures were reoptimized using DFT methods (B3LYP-GD3/6-311++G(d,p)), and the six low-energy structures, those below 1000 cm^–1^, belong to the two configurations shown in Figure 2. In configuration I, rings *b* and *c* (see Scheme 1) adopt a chair conformation, while ring *a* takes a half-chair disposition. Ring *a* is almost in the same plane as rings *b*, *c*, and *d*, and the molecule adopts an extended form. Configuration II is in a semifolded arrangement shown in Figure 2b, with the *a* ring almost perpendicular to the *b*–*c*–*d* rings’ plane. The three lowest-energy conformers of either configuration differ in the hydroxyl group’s orientation for each configuration, as indicated in Figure S1. As shown in Table 1, the three conformers belonging to configuration I are considerably more stable than those belonging to configuration II, resulting in extra stability of 600 cm^–1^.

**Figure 2:**
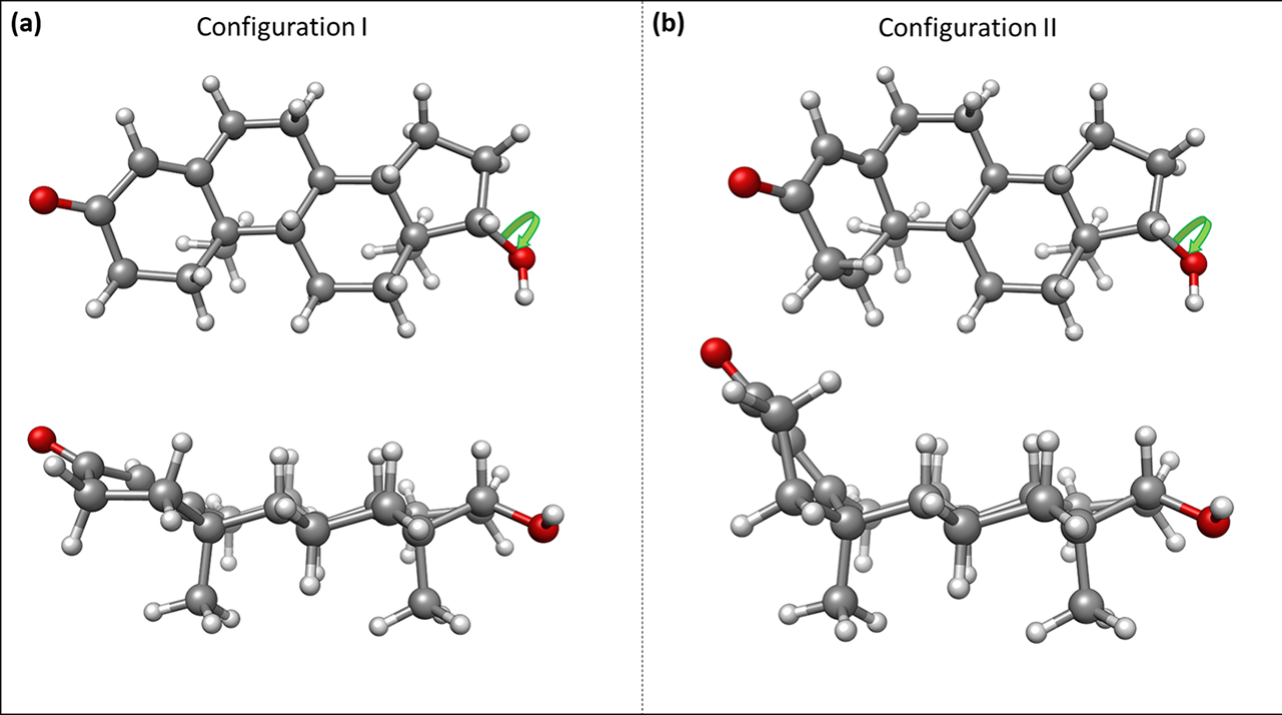
A comparison between the two plausible configurations of testosterone. A top and side view of (a) structure 1 within the extended configuration and (b) structure 4 within the semifolded configuration. For each configuration, three conformers are possible differing in the hydroxyl group’s orientation, as indicated by the green arrow.

We used two different indicators for conformer identification: rotational constants and dipole moment components. The rotational constants provide information on the rotamer’s mass distribution and are fundamental in obtaining conformational structures. The comparison of Table 1 between the experimental rotational constants with the theoretically predicted values unambiguously assigns the observed rotamer to one of the three conformers of configuration I, i.e., the extended form. Unfortunately, the values of the rotational constants are not able to discern between the three structures 1, 2, and 3 that differ only in the orientation of the terminal hydroxyl group. This small difference does not cause a significant change in the mass distribution and, consequently, in the rotational constants’ values. The missing information can be obtained from the dipole moment components, which are directly connected with the selection rules and intensity of the observed transitions. We estimate relative intensities of 6.5:1.0:1.5 for the *a*-, *b*-, anc *c*-type transitions, respectively. The intensity of the *a*-type transitions is significantly larger than those of *b-* and *c-*type in good agreement with the dipole moment selection rules predicted for structures 1 and 3 but in sharp contrast to intense *c*-type transitions expected for structure 2, which therefore can be ruled out. The distinction between structures 1 and 3 is not definitive. However, calculations using both DFT and MP2 methods predict structure 1 to be the global minimum and, therefore, is most likely the detected structure of testosterone.

Having deduced a plausible assignment of testosterone’s observed rotamer, we conclude the spectral analysis by explaining why we observe only a single conformer, turning our attention to the properties of a supersonic expansion. By examining the behavior of a variety of molecules, Ruoff et al. noted that, when a low potential energy barrier separates the conformational species, the noble-gas collisions in the initial stages of the expansion provide the energy required for their interconversion.^19,20^ We used theoretical calculations to perform the relaxed potential energy surface (PES) scan shown in Figure 3 by rotating the C–C–O–H dihedral angle. The barriers to hydroxyl conformer interconversion are below 400 cm^–1^, suggesting that neon should be capable of relaxing structure 2 into structure 3 and the latter into structure 1. Nevertheless, at room temperature, testosterone should participate in a dynamic equilibrium of the three conformers.

**Figure 3:**
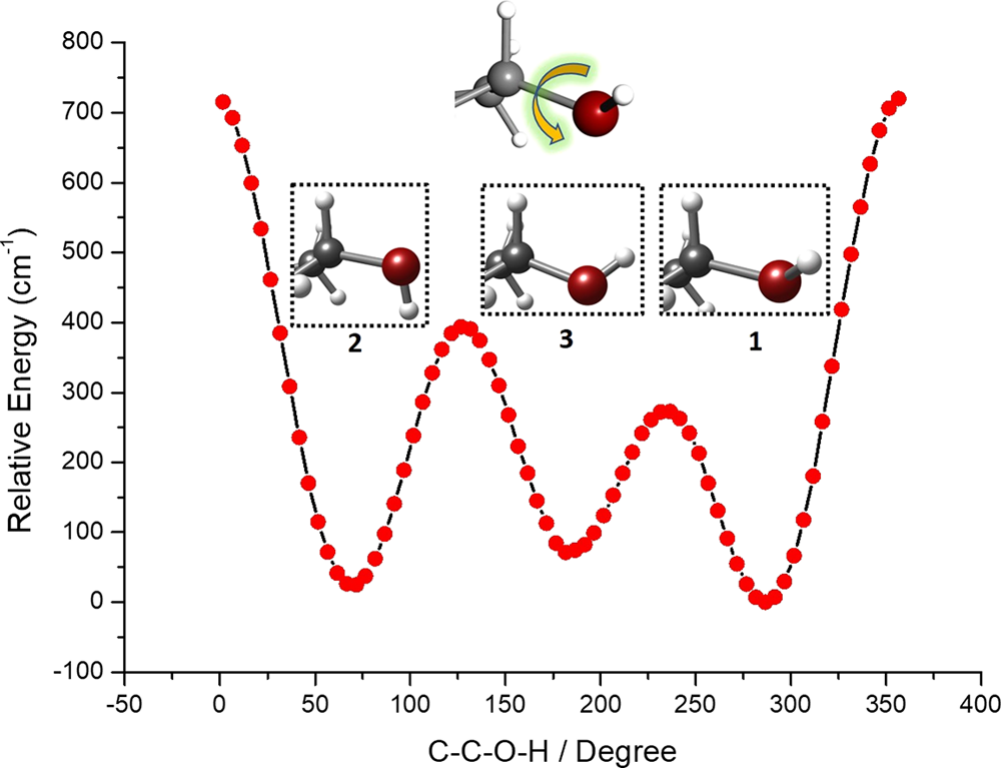
Relaxed PES rotating the C–C–O–H dihedral angle of the three lowest-energy conformers of testosterone within configuration I. The low barrier separating them explains why a single conformer is seen. A close-up view of the hydroxyl group position is shown, while a larger view of the structures can be found in the SI.

We unambiguously determined that the extended form of testosterone is the most stable configuration. Subsequently, this knowledge allows for some conclusions about its biological activity due to the structure–property correspondence. Explanation of epimers’ activity differences is likely to be related to the substituents’ gross spatial orientation rather than to subtle conformational changes in the steroid skeleton.^6^ From our results in Figure 2, it is clear that the semifolded form of testosterone, in which ring *a* is out of plane from the rest of the structure, is not suitable to establish a hydrogen bond between subsequent testosterone molecules. Furthermore, it would not allow direct interactions between stacked molecules, thus precluding van der Waals interactions between different ribbons. The extended form, on the other hand, allows both types of interactions. It is in excellent agreement with the crystal testosterone’s structural arrangement:^6^ The testosterone’s unit cell consists of two extended parallel ribbons of the molecules extended into a sheet. These ribbons are hydrogen-bonded sequentially between the hydroxyl group of a molecule and the next molecule’s ketone group. Strong van der Waals interactions bind both ribbons together, which are in a *head-to-tail* disposition. It is important to note that these results correlate very well with the arrangement observed in steroids in the condensed phase.^21,22^

An interesting comparison is that of testosterone with estradiol. Estradiol is similar to testosterone but with ring *a* differing considerably: the methyl group in ring *a* is lost and it has a hydroxyl group instead of the ketone group, leaving ring *a* as a phenolic ring. This change makes estradiol lose the two possible ring configurations in testosterone, which is now planar. Effectively, the structure of estradiol has a single configuration but six possible conformers that differ in the hydroxyl groups’ orientations. The rotational spectrum of estradiol^11^ shows two predominant structures that are similar to our characterized structure but differing in the two possible orientations of the hydroxyl group in ring *a*. This is another point supporting our assignment. Additionally, a third conformer is found, which is much weaker than expected due to conformational interconversion. It further confirms our assignment and discussion about conformational interconversion. Comparing both steroids is interesting, because the characterized predominant species are very similar but have entirely different biological functionalities. Because the only structural difference is ring *a*, it must be responsible for their ligand–protein binding in different receptors. The variation from a carbonyl to a hydroxyl group probably forces a binding with a receptor capable of a proton donor or proton acceptor, respectively. Additionally, the receptor must be ready to adapt to the dispersive forces or sterical effects caused by the methyl group in testosterone. This comparison is another illustrative example of the importance of the structure–property relationship.

In summary, we have been able to transfer testosterone, a large biomolecule, from its solid into the gas phase using laser ablation techniques. It has allowed us to characterize the structure of a relevant biomolecule such as testosterone by rotational spectroscopy for the first time. This technique is one of the most powerful spectroscopic techniques for structural determination due to the direct relation of shape and spectral position of 404 rotational lines. Interestingly, out of the two possible configurations, the extended form is the predominant one. Supersonic-jet analytical techniques combined with laser ablation can be used to determine a sample’s composition without dissolving the sample in a suitable medium that would usually require chemical manipulations. Additionally, there is no challenging or tedious preparation of the sample requiring lengthy analysis times. As we show, the sensitivity reached using our experimental procedure is sufficient to detect large biomolecules or common pharmaceuticals with unrivaled structural determination.

## Supporting Information Available

Detailed experimental and theoretical section; detailed view of the configurations of testosterone (Figure S1); measured frequencies for the 404 transitions of the testosterone’s detected rotamer (Table S1) (PDF)

## References

[ref1] Mooradian A. D.; Morley J. E.; Korenman S. G. Biological Actions of Androgens. Endocr. Rev. 1987, 8, 1–28. 10.1210/edrv-8-1-1. 3549275

[ref2] Rommerts F. F. G. Testosterone: An Overview of Biosynthesis, Transport, Metabolism and Nongenomic Actions. Testosterone 1998, 1 10.1007/978-3-642-72185-4_1.

[ref3] Lehninger A.; Nelson D.; Cox M. Lehninger Principles of Biochemistry, 5th ed.; W. H. Freeman: New York, 2008.

[ref4] Park Y.; Helms V. On the Derivation of Propensity Scales for Predicting Exposed Transmembrane Residues of Helical Membrane Proteins. Bioinformatics 2007, 23, 701–708. 10.1093/bioinformatics/btl653. 17237049

[ref5] Hayamizu K.; Kamo O. Complete Assignments of the 1H and 13C NMR Spectra of Testosterone and 17α-methyltestosterone and the 1H Parameters Obtained from 600 MHz Spectra. Magn. Reson. Chem. 1990, 28, 250–256. 10.1002/mrc.1260280311.

[ref6] Roberts P. J.; Pettersen R. C.; Sheldrick G. M.; Isaacs N. W.; Kennard O. Crystal and Molecular Structure of 17β-Hydroxyandrost-4-En-3-One (Testosterone). J. Chem. Soc., Perkin Trans. 2 1973, 2, 1978–1984. 10.1039/P29730001978.

[ref7] Robertson E. G.; Simons J. P. Getting into Shape: Conformational and Supramolecular Landscapes in Small Biomolecules and Their Hydrated Clusters. Phys. Chem. Chem. Phys. 2001, 3, 1–18. 10.1039/b008225m.

[ref8] De Vries M. S.; Hobza P. Gas-Phase Spectroscopy of Biomolecular Building Blocks DFT: Density-Functional Theory. Annu. Rev. Phys. Chem. 2007, 58, 585–612. 10.1146/annurev.physchem.57.032905.104722. 17291183

[ref9] Oomens J.; Steill J. D.; Redlich B. Gas-Phase IR of Deprotonated Amino Acids. J. Am. Chem. Soc. 2009, 131, 4310–4319. 10.1021/ja807615v. 19267428

[ref10] Caminati W. Nucleic Acid Bases in the Gas Phase. Angew. Chem., Int. Ed. 2009, 48, 9030–9033. 10.1002/anie.200902993. 19856354

[ref11] Zinn S.; Schnell M. Flexibility at the Fringes: Conformations of the Steroid Hormone β-Estradiol. ChemPhysChem 2018, 19, 2915–2920. 10.1002/cphc.201800647. 30055108

[ref12] Alonso E. R.; León I.; Alonso J. L. The Role of the Intramolecular Interactions in the Structural Behavior of Biomolecules: Insights from Rotational Spectroscopy. Intra- and Intermolecular Interactions Between Non-covalently Bonded Species 2021, 93 10.1016/B978-0-12-817586-6.00004-9.

[ref13] Mata S.; Pena I.; Cabezas C.; López J. C.; Alonso J. L. A Broadband Fourier-Transform Microwave Spectrometer with Laser Ablation Source: The Rotational Spectrum of Nicotinic Acid. J. Mol. Spectrosc. 2012, 280, 91–96. 10.1016/j.jms.2012.08.004.

[ref14] Cabezas C.; Varela M.; Alonso J. L. The Structure of the Elusive Simplest Dipeptide Gly-Gly. Angew. Chem. 2017, 129, 6520–6525. 10.1002/ange.201702425. 28455904

[ref15] León I.; Alonso E. R.; Cabezas C.; Mata S.; Alonso J. L. Unveiling the N→π* Interactions in Dipeptides. Commun. Chem. 2019, 2, 3 10.1038/s42004-018-0103-2.

[ref16] Peña I.; Cabezas C.; Alonso J. L. The Nucleoside Uridine Isolated in the Gas Phase. Angew. Chem., Int. Ed. 2015, 54, 2991–2994. 10.1002/anie.201412460. PMC4892345 25683559

[ref17] Bermúdez C.; Mata S.; Cabezas C.; Alonso J. L. Tautomerism in Neutral Histidine. Angew. Chem., Int. Ed. 2014, 53, 11015–11018. 10.1002/anie.201405347. 25146969

[ref18] Pickett H. M. The Fitting and Prediction of Vibration-Rotation Spectra with Spin Interactions. J. Mol. Spectrosc. 1991, 148, 371–377. 10.1016/0022-2852(91)90393-O.

[ref19] Ruoff R. S.; Klots T. D.; Emilsson T.; Gutowsky H. S. Relaxation of Conformers and Isomers in Seeded Supersonic Jets of Inert Gases. J. Chem. Phys. 1990, 93, 3142–3150. 10.1063/1.458848.

[ref20] Godfrey P. D.; Brown R. D.; Rodgers F. M. The Missing Conformers of Glycine and Alanine: Relaxation in Seeded Supersonic Jets. J. Mol. Struct. 1996, 376, 65–81. 10.1016/0022-2860(95)09065-7.

[ref21] Herbert R. B. Terpenes and Steroids. The Biosynthesis of Secondary Metabolites 1981, 50 10.1007/978-94-009-5833-3_4.

[ref22] Weeks C. M.; Cooper A.; Norton D. A.; Hauptman H.; Fisher J. The Crystal and Molecular Structures of 5α-Androstan-3β-Ol-17-One and 5β-Androstane-3α,L7β-Diol. Acta Crystallogr., Sect. B: Struct. Crystallogr. Cryst. Chem. 1971, 27, 1562–1572. 10.1107/S0567740871004382.

