# Supplementary material for: Shape of Testosterone
Source: J Phys Chem Lett. 2021 Jul 20;12(29):6983–7. doi: 10.1021/acs.jpclett.1c01743 (PMC8327310; doi:10.1021/acs.jpclett.1c01743)
Supplement: Supplementary file 1 — jz1c01743_si_001.pdf [file jz1c01743_si_001.pdf]

Supporting information for:

The Shape of Testosterone

I. León,*[a] E. R. Alonso,[b][c] S. Mata,[a] J. L. Alonso [a]

[a]Grupo de Espectroscopía Molecular (GEM), Edificio Quifima, Laboratorios de Espectroscopia y Bioespectroscopia, Unidad Asociada CSIC, Parque Científico UVa, Universidad de Valladolid, 47011 Valladolid, Spain.

[b]Instituto Biofisika (UPV/EHU, CSIC), University of the Basque Country, 48940, Leioa, Spain.

[b]Departamento de Química Física, Facultad de Ciencia y Tecnología, Universidad del País Vasco, Barrio Sarriena s/n, 48940, Leioa, Spain.

*Corresponding Author:

Iker León Ona, ileon@uva.es

phones: +34 983 186344 / +34 983 186349

web: http://www.gem.uva.es/

METHODS

Experimental details

A commercial sample of testosterone (Cymit, >98%) was used without any further purification. A solid rod was prepared by pressing the compound's fine powder mixed with a small amount of commercial binder and was placed in the ablation nozzle. A picosecond Nd:YAG laser (20 mJ per pulse, 20 ps pulse width) was used as a vaporization tool. Products of the laser ablation were supersonically expanded using the flow of carrier gas (Ne, 8 bar) and characterized by chirped-pulse Fourier transform microwave spectroscopy (LA-CP-FTMW), using a recent constructed instrument[1] dedicated to maximize its performance from 1.5 to 8 GHz. This newly built spectrometer is ideal to record the rotational spectrum of large molecules such as testosterone.

Theoretical Modeling

The conformational space of testosterone was first explored using fast molecular mechanics methods. *MMFFs* forcefield and two search algorithms: the "Large scales Low Mode" (which uses frequency modes to create new structures) and a Monte Carlo-based search, as implemented in Macromodel[2] was used, obtaining a total of 8 structures using a 30 kJ/mol energy cut off.

Geometry optimizations testosterone were done using Gaussian suite programs.[3] The model of choice for the main work was the density functional theory (DFT) methods[4–6] with Grimme dispersion corrections (GD3),[7] with the Pople's 6-311++G(d,p) basis set[8]. Frequency calculations were also computed to ensure that the optimized geometries are true minima and to calculate the Gibbs free energies.

Fig. S1 A comparison between the two plausible configurations of testosterone. A side view of (a) structure 1 and (b) structure 4 highlighting the various dispositions of the *a* ring. For each configuration, there are three conformers differing in the hydroxyl group's orientation.

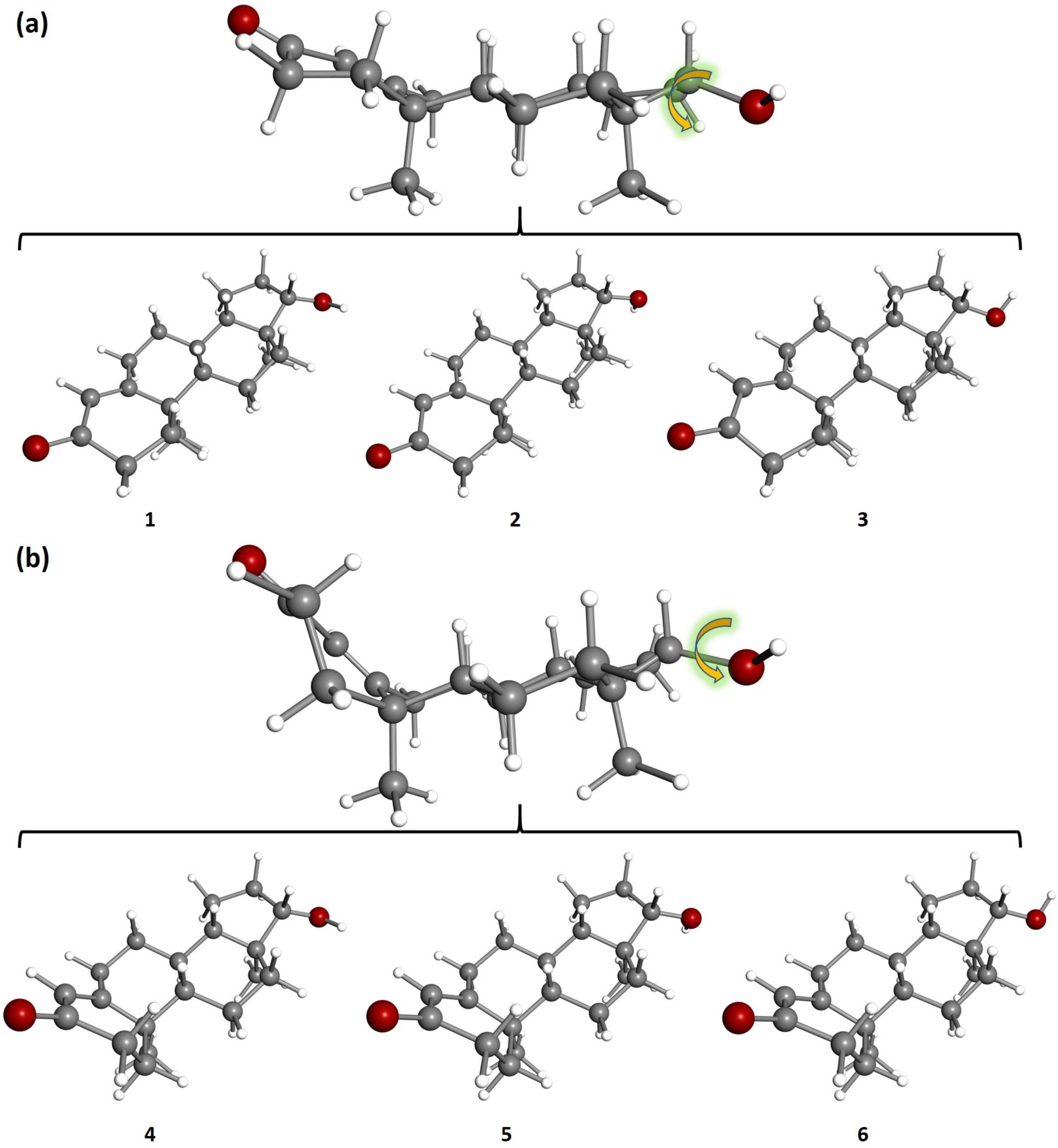

Table S1. Measured frequencies and residuals (in MHz) for the transitions of the testosterone's detected rotamer.

J´	$K´_{-1}$	$K´_{+1}$	J´´	$K´´_{-1}$	$K´´_{+1}$	$\nu_{obs.}$	$\nu_{obs.}-\nu_{cal.}$
5	0	5	4	0	4	1606.595	-0.008
5	2	4	4	2	3	1611.342	-0.007
5	2	3	4	2	2	1616.718	0.020
5	1	4	4	1	3	1648.112	0.002
6	1	6	5	1	5	1887.432	0.006
6	0	6	5	0	5	1925.030	-0.003
6	2	5	5	2	4	1933.104	-0.020
6	5	1	5	5	0	1934.981	-0.030
6	4	2	5	4	1	1935.251	0.002
6	4	3	5	4	2	1935.251	0.003
6	3	4	5	3	3	1935.704	-0.030
6	3	3	5	3	2	1935.931	0.030
6	2	4	5	2	3	1942.420	-0.009
6	1	5	5	1	4	1976.955	0.008
4	1	3	3	0	3	1989.068	-0.008
7	1	7	6	1	6	2201.087	0.020
7	0	7	6	0	6	2241.973	0.006
7	2	6	6	2	5	2254.643	0.005
7	5	3	6	5	2	2257.659	-0.020
7	5	2	6	5	1	2257.659	-0.020
7	4	3	6	4	2	2258.071	0.008
7	4	4	6	4	3	2258.071	0.010
7	3	5	6	3	4	2258.775	0.005
7	3	4	6	3	3	2259.143	-0.006
7	2	5	6	2	4	2269.400	0.007
7	1	6	6	1	5	2305.327	-0.001
6	1	6	5	0	5	2407.016	0.020
2	2	0	1	1	0	2509.989	-0.040
8	1	8	7	1	7	2514.324	0.001
8	0	8	7	0	7	2557.261	0.005
8	2	7	7	2	6	2575.837	0.001
8	7	1	7	7	0	2579.939	-0.010
8	6	2	7	6	1	2580.131	0.004
8	5	3	7	5	2	2580.444	0.020
8	5	4	7	5	3	2580.444	0.020
8	4	4	7	4	3	2581.005	0.001
8	4	5	7	4	4	2581.005	0.009
8	3	6	7	3	5	2581.975	0.009
8	3	5	7	3	4	2582.733	0.010
8	2	6	7	2	5	2597.685	-0.001
7	1	7	6	0	6	2683.045	0.010

Table S1. Cont.

J´	K´$_{-1}$	K´$_{+1}$	J´´	K´´$_{-1}$	K´´$_{+1}$	$\nu_{obs.}$	$\nu_{obs.}$-$\nu_{cal.}$
6	1	5	5	0	5	2720.653	0.001
3	2	1	2	1	1	2818.576	0.030
9	1	9	8	1	8	2827.164	0.005
9	0	9	8	0	8	2870.828	-0.001
9	2	8	8	2	7	2896.682	0.002
9	7	2	8	7	1	2902.618	0.030
9	6	4	8	6	3	2902.863	0.020
9	6	3	8	6	2	2902.863	0.020
9	5	4	8	5	3	2903.274	0.008
9	5	5	8	5	4	2903.274	0.008
9	4	5	8	4	4	2904.091	-0.003
9	4	6	8	4	5	2904.091	0.020
9	3	7	8	3	6	2905.318	0.009
9	3	6	8	3	5	2906.702	0.010
9	2	7	8	2	6	2927.337	0.004
9	1	8	8	1	7	2960.310	0.001
8	3	6	8	2	6	3080.756	0.010
7	3	5	7	2	5	3096.457	-0.006
7	3	4	7	2	5	3097.060	-0.040
7	1	6	6	0	6	3100.945	-0.001
6	3	4	6	2	4	3107.086	0.001
6	3	3	6	2	4	3107.378	0.040
5	3	3	5	2	3	3113.810	0.020
4	2	3	3	1	2	3117.146	0.020
4	2	2	3	1	2	3121.158	0.008
7	3	5	7	2	6	3129.901	0.020
7	3	4	7	2	6	3130.514	-0.001
8	3	5	8	2	7	3137.402	0.002
10	1	10	9	1	9	3139.562	0.005
10	0	10	9	0	9	3182.704	0.001
4	2	3	3	1	3	3206.830	0.020
10	2	9	9	2	8	3217.121	-0.005
9	1	9	8	0	8	3225.273	-0.020
10	7	4	9	7	3	3225.273	-0.001
10	7	3	9	7	2	3225.273	-0.001
10	6	5	9	6	4	3225.623	0.004
10	6	4	9	6	3	3225.623	0.004
10	5	5	9	5	4	3226.204	-0.002
10	5	6	9	5	5	3226.204	-0.002
10	4	6	9	4	5	3227.325	-0.030
10	4	7	9	4	6	3227.325	0.020

Table S1. Cont.

J´	$K´_{-1}$	$K´_{+1}$	J´´	$K´´_{-1}$	$K´´_{+1}$	$\nu_{obs.}$	$\nu_{obs.}-\nu_{cal.}$
10	3	8	9	3	7	3228.799	0.010
10	3	7	9	3	6	3231.153	0.005
10	2	8	9	2	7	3258.269	0.001
10	1	9	9	1	8	3286.676	-0.002
5	2	4	4	1	3	3409.598	0.030
5	2	3	4	1	3	3418.946	0.010
11	1	11	10	1	10	3451.509	0.001
8	1	7	7	0	7	3492.121	-0.009
11	0	11	10	0	10	3492.992	-0.001
10	1	10	9	0	9	3494.026	0.009
11	2	10	10	2	9	3537.129	-0.005
11	8	4	10	8	3	3547.722	0.002
11	8	3	10	8	2	3547.722	0.002
11	7	5	10	7	4	3548.029	0.010
11	7	4	10	7	3	3548.029	0.010
11	6	6	10	6	5	3548.479	0.002
11	6	5	10	6	4	3548.479	0.002
11	5	6	10	5	5	3549.267	0.009
11	5	7	10	5	6	3549.267	0.010
11	4	7	10	4	6	3550.760	-0.040
11	4	8	10	4	7	3550.760	0.060
11	3	9	10	3	8	3552.384	0.005
11	3	8	10	3	7	3556.208	0.020
5	2	4	4	1	4	3559.034	0.003
11	2	9	10	2	8	3590.328	0.001
11	1	10	10	1	9	3612.124	-0.001
6	2	4	5	1	4	3713.266	0.010
11	1	11	10	0	10	3762.780	-0.040
12	1	12	11	1	11	3763.027	0.010
12	0	12	11	0	11	3801.900	0.002
12	2	11	11	2	10	3856.665	0.001
12	8	5	11	8	4	3870.399	-0.040
12	8	4	11	8	3	3870.399	-0.040
12	7	6	11	7	5	3870.829	0.010
12	7	5	11	7	4	3870.829	0.010
12	6	7	11	6	6	3871.431	0.010
12	6	6	11	6	5	3871.431	0.010
12	5	7	11	5	6	3872.440	0.006
12	5	8	11	5	7	3872.440	0.009
12	4	9	11	4	8	3874.284	-0.004
12	4	8	11	4	7	3874.493	0.030

Table S1. Cont.

J´	$K´_{-1}$	$K´_{+1}$	J´´	$K´´_{-1}$	$K´´_{+1}$	$\nu_{obs.}$	$\nu_{obs.}-\nu_{cal.}$
12	3	10	11	3	9	3876.050	0.002
12	3	9	11	3	8	3881.930	-0.003
9	1	8	8	0	8	3895.160	-0.020
6	2	5	5	1	5	3918.733	0.007
12	2	10	11	2	9	3923.251	-0.006
12	1	11	11	1	10	3936.495	-0.006
6	2	4	5	1	5	3937.472	0.080
7	2	6	6	1	5	3972.294	0.010
7	2	5	6	1	5	4005.708	0.008
12	1	12	11	0	11	4032.835	-0.009
13	1	13	12	1	12	4074.082	0.005
3	3	0	2	2	0	4087.795	-0.030
3	3	1	2	2	1	4088.131	0.040
13	0	13	12	0	12	4109.681	0.002
13	2	12	12	2	11	4175.680	-0.001
13	10	4	12	10	3	4192.732	0.100
13	9	4	12	9	3	4192.895	0.020
13	8	6	12	8	5	4193.216	0.010
13	8	5	12	8	4	4193.216	0.010
13	7	7	12	7	6	4193.703	0.010
13	7	6	12	7	5	4193.703	0.010
13	6	8	12	6	7	4194.480	0.030
13	6	7	12	6	6	4194.480	0.030
13	5	8	12	5	7	4195.750	0.004
13	5	9	12	5	8	4195.750	0.009
13	4	10	12	4	9	4198.066	0.003
13	4	9	12	4	8	4198.381	0.001
13	3	11	12	3	10	4199.757	0.003
13	3	10	12	3	9	4208.495	-0.002
13	1	12	12	1	11	4259.652	-0.001
7	2	6	6	1	6	4285.961	0.020
8	2	6	7	1	6	4298.072	0.010
13	1	13	12	0	12	4305.072	0.050
14	1	14	13	1	13	4384.714	-0.002
14	0	14	13	0	13	4416.620	0.003
14	2	13	13	2	12	4494.151	0.002
9	2	8	8	1	7	4506.316	-0.002
14	10	5	13	10	4	4515.386	0.070
14	10	4	13	10	3	4515.386	0.070
14	9	6	13	9	5	4515.628	0.020
14	9	5	13	9	4	4515.628	0.020

Table S1. Cont.

J´	K´$_{-1}$	K´$_{+1}$	J´´	K´´$_{-1}$	K´´$_{+1}$	$\nu_{obs.}$	$\nu_{obs.}-\nu_{cal.}$
14	8	7	13	8	6	4516.031	0.005
14	8	6	13	8	5	4516.031	0.005
14	7	8	13	7	7	4516.654	0.020
14	7	7	13	7	6	4516.654	0.020
14	6	8	13	6	7	4517.595	0.008
14	6	9	13	6	8	4517.595	0.008
14	5	9	13	5	8	4519.192	-0.010
14	5	10	13	5	9	4519.192	-0.003
14	4	11	13	4	10	4522.036	0.001
14	4	10	13	4	9	4522.571	-0.002
14	3	12	13	3	11	4523.448	0.001
14	3	11	13	3	10	4536.004	-0.004
14	1	14	13	0	13	4580.030	-0.030
14	1	13	13	1	12	4581.408	-0.010
14	2	12	13	2	11	4590.436	-0.008
9	2	7	8	1	7	4592.248	0.008
8	2	7	7	1	7	4660.691	-0.010
15	1	15	14	1	14	4694.956	0.004
15	0	15	14	0	14	4722.989	0.002
5	3	2	4	2	2	4730.541	-0.020
5	3	3	4	2	2	4730.541	0.060
5	3	2	4	2	3	4734.530	-0.050
5	3	3	4	2	3	4734.530	0.030
10	2	9	9	1	8	4763.132	-0.002
15	2	14	14	2	13	4812.035	-0.005
15	10	6	14	10	5	4838.075	0.040
15	10	5	14	10	4	4838.075	0.040
15	9	7	14	9	6	4838.417	0.020
15	9	6	14	9	5	4838.417	0.020
15	8	8	14	8	7	4838.932	0.020
15	8	7	14	8	6	4838.932	0.020
15	7	9	14	7	8	4839.670	0.010
15	7	8	14	7	7	4839.670	0.010
15	6	9	14	6	8	4840.839	0.010
15	6	10	14	6	9	4840.839	0.010
15	5	10	14	5	9	4842.822	-0.006
15	5	11	14	5	10	4842.822	0.020
15	4	12	14	4	11	4846.210	0.001
15	4	11	14	4	10	4847.075	-0.010
15	3	13	14	3	12	4847.076	0.001
15	3	12	14	3	11	4864.587	-0.002

Table S1. Cont.

J´	K'_{-1}	K'_{+1}	J´´	K''_{-1}	K''_{+1}	$\nu_{obs.}$	$\nu_{obs.}-\nu_{cal.}$
10	2	8	9	1	8	4890.208	0.008
15	1	14	14	1	13	4901.632	-0.006
15	2	13	14	2	12	4924.003	-0.010
16	1	16	15	1	15	5004.818	0.006
16	0	16	15	0	15	5029.036	0.007
9	2	8	8	1	8	5043.070	0.008
6	3	4	5	2	3	5049.528	0.010
6	3	3	5	2	3	5049.766	-0.002
6	3	4	5	2	4	5058.879	-0.001
6	3	3	5	2	4	5059.101	-0.030
16	2	15	15	2	14	5129.325	-0.003
16	10	6	15	10	5	5160.843	0.050
16	8	9	15	8	8	5161.879	0.020
16	8	8	15	8	7	5161.879	0.020
16	7	10	15	7	9	5162.779	0.010
16	7	9	15	7	8	5162.779	0.010
16	5	11	15	5	10	5166.600	-0.020
16	5	12	15	5	11	5166.600	0.020
16	4	13	15	4	12	5170.579	-0.004
16	3	14	15	3	13	5170.579	0.004
16	4	12	15	4	11	5171.959	-0.006
12	1	11	11	0	11	5183.942	-0.020
11	2	9	10	1	9	5193.845	-0.003
16	3	13	15	3	12	5194.353	0.002
16	1	15	15	1	14	5220.157	-0.010
16	2	14	15	2	13	5257.122	-0.020
17	1	17	16	1	16	5314.329	0.002
17	0	17	16	0	16	5334.948	0.020
7	3	5	6	2	4	5365.837	-0.020
7	3	4	6	2	4	5366.503	0.020
7	3	5	6	2	5	5384.532	0.010
7	3	4	6	2	5	5385.166	0.010
10	2	9	9	1	9	5433.028	-0.002
17	2	16	16	2	15	5445.970	-0.020
17	9	9	16	9	8	5484.139	0.010
17	9	8	16	9	7	5484.139	0.010
17	8	10	16	8	9	5484.888	0.020
17	8	9	16	8	8	5484.888	0.020
17	7	11	16	7	10	5485.969	0.007
17	7	10	16	7	9	5485.969	0.007
17	6	11	16	6	10	5487.672	0.002

Table S1. Cont.

J´	K´$_{-1}$	K´$_{+1}$	J´´	K´´$_{-1}$	K´´$_{+1}$	$\nu_{obs.}$	$\nu_{obs.}$-$\nu_{cal.}$
17	6	12	16	6	11	5487.672	0.003
17	5	12	16	5	11	5490.585	-0.020
17	5	13	16	5	12	5490.585	0.050
17	3	15	16	3	14	5493.879	-0.007
17	4	14	16	4	13	5495.144	-0.006
12	2	10	11	1	10	5504.979	-0.002
17	3	14	16	3	13	5525.370	-0.007
17	1	16	16	1	15	5536.881	-0.004
17	2	15	16	2	14	5589.554	-0.010
13	5	9	13	4	9	5607.408	-0.006
13	5	9	13	4	10	5608.059	-0.030
12	5	7	12	4	8	5610.027	-0.030
12	5	8	12	4	8	5610.027	-0.030
12	5	7	12	4	9	5610.447	0.030
12	5	8	12	4	9	5610.447	0.030
11	5	6	11	4	7	5612.009	-0.080
11	5	7	11	4	7	5612.009	-0.080
11	5	6	11	4	8	5612.342	0.070
11	5	7	11	4	8	5612.342	0.070
10	5	5	10	4	7	5613.661	-0.060
10	5	6	10	4	7	5613.661	-0.050
10	5	5	10	4	6	5613.661	0.030
10	5	6	10	4	6	5613.661	0.030
9	5	4	9	4	6	5614.783	-0.030
9	5	5	9	4	6	5614.783	-0.030
9	5	4	9	4	5	5614.783	0.005
9	5	5	9	4	5	5614.783	0.006
8	5	3	8	4	5	5615.593	-0.030
8	5	4	8	4	5	5615.593	-0.030
8	5	4	8	4	4	5615.593	-0.010
8	5	3	8	4	4	5615.593	-0.010
7	5	2	7	4	4	5616.171	-0.020
7	5	3	7	4	4	5616.171	-0.020
7	5	3	7	4	3	5616.171	-0.010
7	5	2	7	4	3	5616.171	-0.010
6	5	2	6	4	2	5616.555	-0.010
6	5	1	6	4	2	5616.555	-0.010
18	1	18	17	1	17	5623.527	-0.002
18	0	18	17	0	17	5640.828	-0.004
4	4	0	3	3	0	5658.657	-0.001
8	3	6	7	2	5	5678.429	0.001

Table S1. Cont.

J´	K´$_{-1}$	K´$_{+1}$	J´´	K´´$_{-1}$	K´´$_{+1}$	$\nu_{obs.}$	$\nu_{obs.}$-$\nu_{cal.}$
8	3	5	7	2	5	5679.811	-0.005
8	3	6	7	2	6	5711.854	0.005
14	2	13	13	1	12	5731.814	0.009
18	2	17	17	2	16	5762.009	-0.006
18	8	11	17	8	10	5807.995	0.040
18	8	10	17	8	9	5807.995	0.040
18	7	12	17	7	11	5809.255	0.001
18	7	11	17	7	10	5809.255	0.001
18	6	12	17	6	11	5811.284	-0.001
18	6	13	17	6	12	5811.284	0.002
18	4	15	17	4	14	5819.897	-0.001
18	4	14	17	4	13	5823.036	-0.003
13	2	11	12	1	11	5825.235	0.010
11	2	10	10	1	10	5830.598	-0.008
18	1	17	17	1	16	5851.713	-0.001
18	3	15	17	3	14	5857.696	-0.010
18	2	16	17	2	15	5921.044	-0.003
19	1	19	18	1	18	5932.440	-0.010
19	0	19	18	0	18	5946.817	-0.001
5	4	1	4	3	1	5981.099	0.001
9	3	7	8	2	6	5986.060	0.008
9	3	6	8	2	6	5988.843	0.020
9	3	7	8	2	7	6041.330	0.008
19	2	18	18	2	17	6077.394	0.001
19	11	9	18	11	8	6128.848	0.040
19	11	8	18	11	7	6128.848	0.040
19	10	10	18	10	9	6129.372	0.020
19	10	9	18	10	8	6129.372	0.020
19	9	11	18	9	10	6130.111	0.030
19	9	10	18	9	9	6130.111	0.030
19	8	12	18	8	11	6131.127	0.006
19	8	11	18	8	10	6131.127	0.006
19	7	12	18	7	11	6132.657	0.009
19	6	14	18	6	13	6135.039	0.003
19	3	17	18	3	16	6139.670	-0.006
19	4	16	18	4	15	6144.808	-0.001
19	4	15	18	4	14	6149.357	-0.010
14	2	12	13	1	12	6156.021	0.006
19	1	18	18	1	17	6164.621	-0.020
19	3	16	18	3	15	6191.313	-0.004
20	1	20	19	1	19	6241.123	-0.008

Table S1. Cont.

J´	K´$_{-1}$	K´$_{+1}$	J´´	K´´$_{-1}$	K´´$_{+1}$	$\nu_{obs.}$	$\nu_{obs.}-\nu_{cal.}$
19	2	17	18	2	16	6251.393	-0.010
20	0	20	19	0	19	6252.936	-0.003
10	3	8	9	2	7	6287.478	-0.030
10	3	7	9	2	7	6292.651	0.010
6	4	2	5	3	2	6303.487	0.040
10	3	8	9	2	8	6373.432	0.002
20	11	10	19	11	9	6451.672	0.007
20	11	9	19	11	8	6451.672	0.007
20	10	11	19	10	10	6452.276	-0.020
20	10	10	19	10	9	6452.276	-0.020
20	9	12	19	9	11	6453.226	0.070
20	9	11	19	9	10	6453.226	0.070
20	8	13	19	8	12	6454.366	-0.002
20	8	12	19	8	11	6454.366	-0.002
20	7	13	19	7	12	6456.151	0.001
20	7	14	19	7	13	6456.151	0.001
20	6	14	19	6	13	6458.932	-0.010
20	6	15	19	6	14	6458.932	-0.004
20	3	18	19	3	17	6462.013	-0.010
20	5	16	19	5	15	6463.573	0.020
20	5	15	19	5	14	6463.857	0.001
20	4	17	19	4	16	6469.869	0.010
20	1	19	19	1	18	6475.702	-0.005
20	4	16	19	4	15	6476.341	0.002
21	1	21	20	1	20	6549.567	-0.030
20	2	18	19	2	17	6580.452	-0.020
21	2	20	20	2	19	6706.190	-0.010
11	3	9	10	2	9	6708.674	-0.009
21	8	14	20	8	13	6777.721	0.020
21	8	13	20	8	12	6777.721	0.020
21	7	14	20	7	13	6779.763	-0.001
21	7	15	20	7	14	6779.763	-0.001
21	6	15	20	6	14	6783.006	-0.001
21	6	16	20	6	15	6783.006	0.010
21	3	19	20	3	18	6783.920	-0.020
21	1	20	20	1	19	6785.045	-0.009
21	4	18	20	4	17	6795.007	-0.001
21	4	17	20	4	16	6804.020	-0.020
22	0	22	21	0	21	6865.666	0.002
21	2	19	20	2	18	6908.096	-0.020
22	2	21	21	2	20	7019.636	-0.020

Table S1. Cont.

J′	K′$_{-1}$	K′$_{+1}$	J′′	K′′$_{-1}$	K′′$_{+1}$	$\nu_{obs.}$	$\nu_{obs.}-\nu_{cal.}$
22	1	21	21	1	20	7092.872	-0.010
22	10	13	21	10	12	7098.394	0.030
22	10	12	21	10	11	7098.394	0.030
22	9	14	21	9	13	7099.500	-0.009
22	9	13	21	9	12	7099.500	-0.009
22	8	15	21	8	14	7101.128	0.006
22	8	14	21	8	13	7101.128	0.006
22	7	15	21	7	14	7103.515	0.020
22	7	16	21	7	15	7103.515	0.020
22	5	18	21	5	17	7113.260	-0.005
22	5	17	21	5	16	7113.960	-0.020
22	4	19	21	4	18	7120.186	-0.040
22	4	18	21	4	17	7132.556	-0.008
23	1	23	22	1	22	7165.974	-0.020
23	0	23	22	0	22	7172.244	-0.020
22	3	19	21	3	18	7198.457	-0.020
5	5	0	4	4	0	7229.342	-0.010
22	2	20	21	2	19	7234.167	-0.020
23	2	22	22	2	21	7332.490	0.001
23	1	22	22	1	21	7399.412	-0.040
23	3	21	22	3	20	7426.207	0.010
23	6	17	22	6	16	7431.614	-0.030
23	6	18	22	6	17	7431.614	0.010
23	5	18	22	5	17	7439.493	-0.009
23	4	20	22	4	19	7445.429	-0.030
24	1	24	23	1	23	7473.958	-0.030
24	0	24	23	0	23	7478.972	-0.030
23	3	20	22	3	19	7535.476	-0.010
6	5	1	5	4	1	7551.788	-0.030
24	2	23	23	2	22	7644.707	-0.030
24	1	23	23	1	22	7705.068	0.020
25	1	25	24	1	24	7781.865	-0.008
25	0	25	24	0	24	7785.821	-0.040
24	4	20	23	4	19	7792.509	-0.040
24	2	22	23	2	21	7881.103	-0.030

REFERENCES:

(1) Bermúdez, C.; Mata, S.; Cabezas, C.; Alonso, J. L. Tautomerism in Neutral Histidine. *Angew. Chemie - Int. Ed.* **2014**, *53*, 11015–11018.

(2) Schrödinger Release 2018-3: Maestro Schrödinger, LLC, New York, NY, 2018. Schrödinger Release 2018-3. Maestro. LLC: New York.

(3) Frisch, M. J.; Trucks, G. W.; Schlegel, H. B.; Scuseria, G. E.; Robb, M. A.; Cheeseman, J. R.; Scalmani, G.; Barone, V.; Mennucci, B.; Petersson, G. A.; Nakatsuji, H.; Caricato, M.; Li, X.; Hratchian, H. P.; Izmaylov, A. F.; Bloino, J.; Zheng, G.; Sonnenberg, J. L.; Hada, M.; Ehara, M.; Toyota, K.; Fukuda, R.; Hasegawa, J.; Ishida, M.; Nakajima, T.; Honda, Y.; Kitao, O.; Nakai, H.; Vreven, T.; Montgomery, J. A.; Jr.; Peralta, J. E.; Ogliaro, F.; Bearpark, M.; Heyd, J. J.; Brothers, E.; Kudin, K. N.; Staroverov, V. N.; Kobayashi, R.; Normand, J.; Raghavachari, K.; Rendell, A. P.; Burant, J. C.; Iyengar, S. S.; Tomasi, J.; Cossi, M.; Rega, N.; Millam, M. J.; Klene, M.; Knox, J. E.; Cross, J. B.; Bakken, V.; Adamo, C.; Jaramillo, J.; Gomperts, R.; Stratmann, R. E.; Yazyev, O.; Austin, A. J.; Cammi, R.; Pomelli, C.; Ochterski, J. W.; Martin, R. L.; Morokuma, K.; Zakrzewski, V. G.; Voth, G. A.; Salvador, P.; Dannenberg, J. J.; Dapprich, S.; Daniels, A. D.; Farkas, Ö.; Foresman, J. B.; Ortiz, J. V.; Cioslowski, J.; Fox, D. J. *Gaussian*; Gaussian 09 Rev. D01., Gaussian, Inc., Wallingford, CT, 2012, see also: http://www.gaussian.com.

(4) Becke, A. D. Density-Functional Exchange-Energy Approximation with Correct Asymptotic Behavior. *Phys. Rev. A* **1988**, *38*, 3098–3100.

(5) Becke, A. D. A New Mixing of Hartree–Fock and Local Density-functional Theories. *J. Chem. Phys.* **1993**, *98* (2), 1372–1377. https://doi.org/10.1063/1.464304.

(6) Zhao, Y.; Truhlar, D. G. Density Functionals for Noncovalent Interaction Energies of Biological Importance. *J. Chem. Theory Comput.* **2007**, *3*, 289–300.

(7) Grimme, S.; Antony, J.; Ehrlich, S.; Krieg, H. A Consistent and Accurate Ab Initio Parametrization of Density Functional Dispersion Correction (DFT-D) for the 94 Elements H-Pu. *J. Chem. Phys.* **2010**, *132* (15).

(8) Frisch, M. J.; Pople, J. A.; Binkley, J. S. Self-Consistent Molecular Orbital Methods 25. Supplementary Functions for Gaussian Basis Sets. *J. Chem. Phys.* **1984**, *80*, 3265–3269.
